# Electrophysiological evidence for the action of a center-surround mechanism on semantic processing in the left hemisphere

**DOI:** 10.3389/fpsyg.2013.00936

**Published:** 2013-12-30

**Authors:** Diana Deacon, John F. Shelley-Tremblay, Walter Ritter, Anna Dynowska

**Affiliations:** ^1^Psychology Department, Bard College at Simon's RockGreat Barrington, MA, USA; ^2^Psychology Department, University of South AlabamaMobile, AL, USA; ^3^Department of Pediatrics, Albert Einstein College of MedicineBronx, NY, USA; ^4^Neurology Department, New York Presbyterian Hospital, Weill Cornell Medical CenterNew York, NY, USA

**Keywords:** event-related potentials, N400, visual half-field, vocabulary acquisition, center-surround inhibition, semantic priming

## Abstract

Physiological evidence was sought for a center-surround attentional mechanism (CSM), which has been proposed to assist in the retrieval of weakly activated items from semantic memory. The CSM operates by facilitating strongly related items in the “center” of the weakly activated area of semantic memory, and inhibiting less strongly related items in its “surround”. In this study weak activation was created by having subjects acquire the meanings of new words to a recall criterion of only 50%. Subjects who attained this approximate criterion level of performance were subsequently included in a semantic priming task, during which ERPs were recorded. Primes were newly learned rare words, and targets were either synonyms, non-synonymously related words, or unrelated words. All stimuli were presented to the RVF/LH (right visual field/left hemisphere) or the LVF/RH (left visual field/right hemisphere). Under RVF/LH stimulation the newly learned word primes produced facilitation on N400 for synonym targets, and inhibition for related targets. No differences were observed under LVF/RH stimulation. The LH thus, supports a CSM, whereby a synonym in the “center” of attention, focused on the newly learned word, is facilitated, whereas a related word in the “surround” is inhibited. The data are consistent with the view of this laboratory that semantic memory is subserved by a spreading activation system in the LH. Also consistent with our view, there was no evidence of spreading activation in the RH. The findings are discussed in the context of additional recent theories of semantic memory. Finally, the adult right hemisphere may require more learning than the LH in order to demonstrate evidence of meaning acquisition.

## Introduction

While priming paradigms have contributed extensively to the empirical testing of language and memory models, most studies have employed familiar words. There is less research pertaining to how a system could be configured to permit the entry of new vocabulary. Theoretically, newly encoded concepts produce weaker activation during retrieval and recognition. The weaker activation produced by the newly encoded concepts must compete with more strongly encoded, familiar items in semantic memory. In order for weakly activated representations to be retrieved from a background of more strongly activated familiar items the signal to noise ratio must somehow be improved. One proposal is that a center-surround mechanism (CSM) improves the signal to noise ratio by selectively inhibiting competing established representations, while facilitating the activation of newly acquired semantic codes (Carr and Dagenbach, 1990; Dagenbach et al., 1990; Dagenbach and Carr, 1994). This theory will be examined in the present study, as well as determining whether the mechanism operates in both cerebral hemispheres.

The impetus for the theory was an unexpected pattern of data obtained in a behavioral semantic priming paradigm (Dagenbach et al., 1989). Participants were required to make lexical decisions to targets following masked primes. Their performance varied as a function of the threshold-setting task^1^ used. When a simple presence-absence detection task was employed, the usual pattern of facilitation was observed for related targets. In the key condition, however, participants performed a semantic similarity judgment task in the threshold-setting phase, following which related words were responded to less quickly than unrelated words. Carr and Dagenbach (1990) replicated and extended these findings, using targets that were repeats of the prime as well as others that were semantically related. As in the first phase of the Dagenbach et al. (1989) study, participants made either detection, or semantic similarity judgments. The authors assumed that the latter task would encourage participants to use primarily semantic information to complete the lexical decision task in phase two. The prediction was that participants would experience difficulty retrieving the weakly activated masked prime. In order to “pull” the weakly activated prime out of a background of other activated related items they would focus their attention on the prime. This attentional allocation would take the form of increased activation for the weak prime, in conjunction with a decrement in activation for more distantly related words. In support of the CSM hypothesis, participants in the semantic decision condition showed facilitation for responses to repetitions of the prime, and inhibition for more distantly related items.

A related study employed weakly activated primes consisting of unmasked, newly learned rare words (Barnhardt et al., 1996). It was assumed that subjects who were still in the process of acquiring the meanings of new words would have difficulty recalling some of their definitions. The words for which the definitions were not recalled would be functionally analogous to masked primes, in that they would have a low level of activation. The low level of activation was expected to engender the use of the attentional CSM. Participants attempted to learn the meanings of highly uncommon words, with the knowledge that their memory would be tested after a short delay. These words and their definitions were presented for only 7 s, in succession, with the effect that they were difficult to remember. The experimenters trained the participants to the point at which they could recall approximately 50% of the definitions of the rare words, in order to establish relatively weak representations of the concepts in semantic memory.

When targets were related to, but not synonymous with the primes, lexical decisions were speeded only for trials on which the prime definitions had been correctly recalled. But, following synonyms of the rare words, responses were speeded, regardless of whether or not the definition of the newly acquired prime was recalled. They interpreted this finding to support the CSM model in which synonyms (Barnhardt et al., 1996), and repeats (Carr and Dagenbach, 1990) are facilitated. In both cases, the neural elements coding for the weakly activated prime and the subsequent target are semantically identical, forming a “center” of common activation. The center of common activation is facilitated. On the other hand, those words that are related, but not semantically identical, are inhibited by the CSM to better differentiate them from the newly learned, weakly activated concept. Unrelated words would fall beyond the edge of the CSM, and hence would be unaffected. The existence of this mechanism has, nevertheless, been questioned. On the basis of behavioral data, it has been suggested that the weakly activated prime prolongs semantic matching between the prime and target, rather than inhibiting activation of competing words during semantic access (see Kahan, 2000).

Using the N400 component of the event-related potential (ERP), it is possible to examine lexical/semantic processing, independently of post-lexical matching. With the exception of one study (Brown and Hagoort, 1993), significant N400 priming effects have been obtained even when stimuli were masked so as to prevent above threshold recognition, which should have prevented the strategy of semantic matching (Schnyer et al., 1997; Deacon et al., 1998; Kiefer, 2002; Misra and Holcomb, 2003). In the present study, the N400 will thus be employed as an index of lexical/semantic processing. The N400 can be elicited by non-words, even when the non-words are not derived from real words (Deacon et al., 2004a). As these stimuli have no meaning, the N400 cannot reflect the accessing of meaning *per se*, but probably reflects the accrual of information necessary to access meaning. We assume a cascade model here, with top down activation from semantic units to orthographic units accounting for semantic effects on the N400 (Deacon et al., 1995, 2004b). In this regard, several more recent proposals are essentially the same as ours (Lau et al., 2008; Grainger and Holcomb, 2009). As manipulations of a number of semantic variables lawfully influence N400 amplitude it, nevertheless, provides a relatively direct measure of semantic as well as lexical processing. The N400 is a negative wave, usually largest at central, temporal, and posterior sites (Kutas and Federmeier, 2009). There are slight variations in its topography, which might depend upon the reference site used (nose or mastoid) or task differences. It is elicited when participants attempt to extract meaning from words or orthographically legal word-like stimuli, and is usually smaller (less negative) when consecutively presented single words are semantically or associatively related, than when they are unrelated (Bentin et al., 1985, 1993; Deacon et al., 1995, 1999). This finding has been interpreted as facilitation, as it accompanies a decrease in reaction time (RT), or improvement in accuracy in most studies. Conversely, the inhibition of behavioral responses has been reported to be accompanied by more negative N400s (Bermeitinger et al., 2008). Bertmeitinger et al. presented category primes that were forward and backward masked. The prime was relatively long in duration compared to some other masking studies, and was rapidly alternated with the backward mask. Flanking letters also appeared on each side of the prime. For example, the German word VOGEL (bird), presented as a masked prime, appeared as “LMVOGELX.” The targets that produced inhibition were associated exemplars of the category prime. Other evidence of inhibition on the N400 has been gleaned from selective attention paradigms. Unattended words generally produce little or no N400 priming, and can produce inhibition (see Deacon and Shelley-Tremblay, 2000 for a review). This study will attempt to verify whether a CSM operates during the acquisition of new vocabulary, and if the mechanism involves inhibition. If a reversed priming effect is found on the N400 in the current paradigm, this will confirm the inhibitory nature of the CSM using a physiological measure.

A further interest of this investigation was to determine if a center-surround arrangement is supported by both hemispheres, or exclusively by the LH. There are two reasons that the literature would predict a LH locus for the CSM. The first concerns differential sensitivity of the two hemispheres to associative and semantic relatedness. The second reason to predict a LH locus for the CSM relates to the greater effect of attentional manipulations on the LH.

Hemispheric differences have been reported in behavioral, ERP and FMRI studies such that priming, as well as overall fMRI activation (adding primed and unprimed conditions together) are usually greater in the LH when pairs of words are associatively related (Deacon et al., 2004b, Experiment 1; Sachs et al., 2008a,b; Sass et al., 2009). By contrast, priming and greater activation are obtained in the RH when words share semantic features, as do most typical category members (Chiarello et al., 1990; Chiarello and Richards, 1992; Deacon et al., 2004b, Experiment 2, Grose-Fifer and Deacon, 2004; Sachs et al., 2008a,b; Sass et al., 2009). Studies on neurological patients with focal lesions confined to one hemisphere also support the contention of differential representation in the two hemispheres (Chiarello and Church, 1986; Villardita, 1987; Welte, 1993; Hagoort et al., 1996; Swaab et al., 1998; Kotz et al., 1999; Kotz and Friederici, 2003). There are of course exceptions. Chiarello et al. (1990) found no behavioral priming in either VF for pure associates. In fact, the RTs were longer for the associated condition than in the neutral condition. Kandhadai and Federmeier (2010) recorded ERPs in a study where the prime was presented foveally and only the target was lateralized. Associative relationships produced significant N400 priming effects that were equivalent across VFs. These results are not surprising as using a central prime with a lateralized target been found to produce equivalent data to a condition where primes and targets were both foveally presented (Burgess and Simpson, 1988). Two laboratories (Abernethy and Coney, 1990; Koivisto and Laine, 1999, 2000; Collins, 2002) have produced behavioral category priming data that are at odds with those just reviewed. The data of Shears and Chiarello (2003) have more recently established that the different pattern of results obtained appear to be due to the use of the go-no-go paradigm. According to these authors this paradigm might tax the very limited controlled processing resources of the RH. Collectively, however, the majority of experiments appear to indicate that the LH has a predilection for processing associative relationships between words, whereas the RH is sensitive to the semantic features that the words share in common.

**Figure 1:**
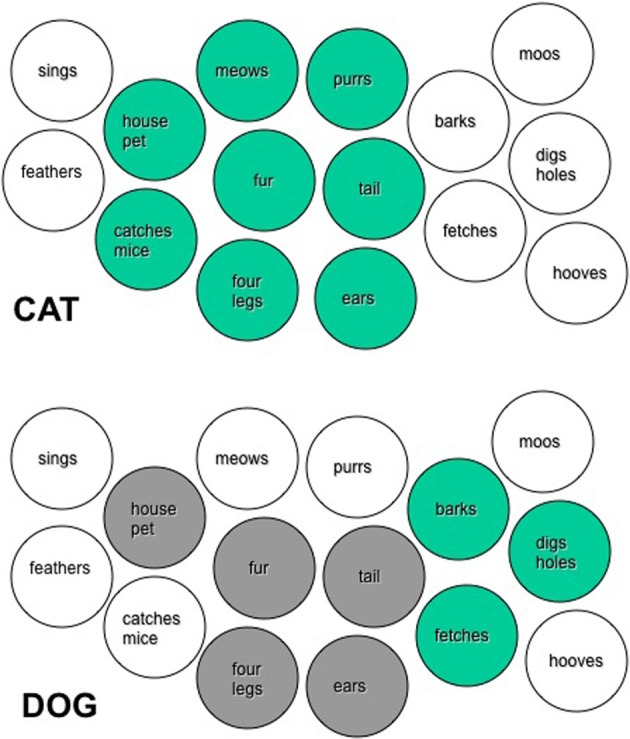
**Schematic depicting the activation of some of the semantic features that would represent the words CAT and DOG, in the distributed system of the RH, during a hypothetical experimental trial, where these words are presented as S1 and S2**. In the **top panel** the circles with green fill correspond to the semantic features that would be activated by CAT. White circles are features of other concepts. In the **bottom panel**, all gray circles represent the features activated by the word DOG. Dark gray circles indicate those that are held in common with CAT, and were thus preactivated before the presentation of DOG. Green circles indicate features that are unique to DOG.

**Figure 2:**
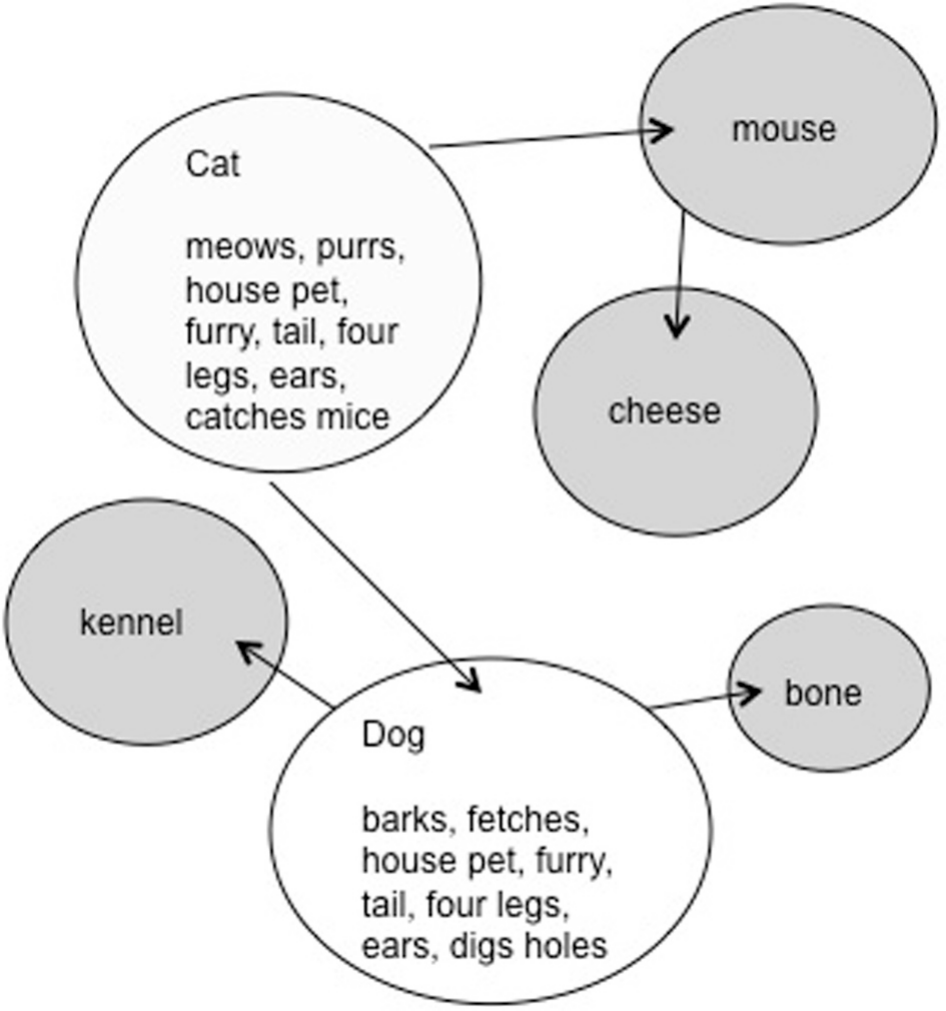
**The activation created by CAT and DOG in the LH semantic system during the same hypothetical experimental trial**. The circles in white represent the activation of the bound features for each gestalt representation. The arrows indicate the associative links between concepts. The gray circles are concepts through which activation has spread via associative links.

This body of research indicates a revision of early automatic spreading activation (ASA) models (e.g., Collins and Loftus, 1975), wherein activation was proposed to spread through associative links between concepts equally on both sides of the brain. The RH semantic memory system cannot rely upon a spreading activation system. Otherwise, an equal number of studies would have found evidence of RH priming by associates, and this has not been the case. While distributed models, wherein the overlap of semantic features activated by a word might account for the RH priming effects (e.g., Masson, 1991), these models do not account for the LH priming effects generated by pure associates, which have no overlapping semantic features. The hemispheric differences noted in the literature that we have reviewed here add further support to the more recently proposed view (Deacon et al., 2004b) that semantic memory operates within a spreading activation system in the LH, whereas there may be no spreading of activation between concepts in the RH (see Figures 1, 2). This distinction was originally tested in three experiments from this laboratory (Deacon et al., 2004b, Experiments 1 and 2; Grose-Fifer and Deacon, 2004). These experiments, and the model that they were designed to test, are relevant in the current context. We will briefly describe the first two of these experiments here in order to clarify the rationale of the present study.

In the first experiment of Deacon et al. (2004a,b), participants were presented with concrete nouns as primes and targets. The primes were either semantically unrelated, or related according to the structure, function, or other attributes of the target. For example, the target BROCCOLI could be preceded by the prime TREE. These items are both plants, share the same global shape, have central trunks with numerous branches, the branches end in leaves or leaf-like inflorescences, and all, or part of each are green. The significance of this stimulus set is that they shared semantic features, but were not associates. We proposed that only the right hemisphere should benefit from primes that are not associates, but share semantic features. This should be the case for a distributed feature network in which common association is not encoded, *per se*, but in which priming is the result of shared features between the prime and target representation. When words sharing features are presented consecutively during an experimental trial, the features of the second item are preactivated by the first (see Figure 1).

The LH should not benefit from such feature overlap as it represents word meanings holistically, with associative links between them (see Figure 2). The LH representations are holistic in the sense that the feature units coding for an item cannot be activated independently. Rather, the multiple features units are bound into a single gestalt unit. The ERP data supported our proposal, in that significant N400 priming was found only for words presented to the LVF/RH.

In the second experiment, concrete nouns were employed that were either unrelated or were related by association. Although associated, the related items did not share semantic features, for example DOG and BONE. Here it was hypothesized that the spreading activation type system, which we proposed to be resident in the LH, would represent associative relationships between items, but would not be sensitive to semantic priming on the basis of feature overlap alone. As predicted, items related strictly through association, produced significant N400 priming in the RVF/LH, but not in the LVF/RH.

In the present study the words will share both features and associations, in order to allow priming to occur in both hemispheres. The RH will benefit from feature overlap and the LH from the associative relationship. An example of this type of word would be CAT and DOG. In the trial illustrated in Figure 1, the word CAT would preactivate many of the same features as DOG (i.e., house pet, fur, tail, four legs) and create facilitation in the RH. The preactivation of the features common to both concepts would decrease the overall time necessary to activate the entire set of semantic features from the orthographic code DOG. The prime CAT presented to the LH will facilitate the target word DOG by virtue of their associative relationship. Spreading activation will travel from CAT to other associated items, including DOG (see Figure 2).

Concerning the CSM model, the experiments just described lead to expectancies regarding hemispheric differences. The CSM is primarily useful in describing how weakly activated items might be accessed in a spreading activation type semantic network, in spite of a poor signal/noise ratio. If a spreading activation system, such as we have proposed, operates in the LH, activation would spread unchecked between associated items, and would decay only as a function of time. In the LH, the CSM should, therefore, assist in the retrieval of weakly activated, newly learned word meanings by dampening the activation of remotely related items, and facilitating the weakly activated new word, as well as synonyms of the new word. If the RH semantic system is distributed and exclusively feature based, there could be no spreading of activation between gestalt representations of concepts. Note that in the absence of gestalt representations and associative links in the RH, there would hypothetically, be no vehicle through which spreading activation could activate entire concepts. Thus, in the RH, the CSM would be superfluous during the acquisition of new vocabulary.

The CSM is proposed to require the allocation of attention. It is therefore of interest that the LH may bear the preponderance of attentional control in semantic processing tasks. Several investigators have concluded that the LH is exclusively responsible for controlled attention driven semantic processing. Early work by Simpson and Burgess (1988) suggested that the LH is responsible for constraining alternative meanings of homographs when they are used to prime sentences. At a long SOA the dominant meanings of homograph primes produced facilitation in both VFs/hemispheres, whereas their subordinate tenses produced inhibition in the LH. No inhibition was observed in the RH. Other evidence of inhibition that was circumscribed to the LH, was reported by Nakagawa (1991). Using masked, foveally presented primes, and lateralized targets, in a lexical decision task, Nakagawa examined hemispheric differences in the processing of three types of words. In comparison to a neutral condition, the data demonstrated an overall pattern of facilitation to targets, when strongly related primes (antonyms) were presented to the RVF/LH, whereas remotely related words, or unrelated words created inhibition. These data are similar to those reported by Dagenbach and colleagues. On the other hand, there was no apparent inhibition for any LVF/RH targets, but rather facilitation of both strong (antonyms) and remotely related items. When Nakagawa modified the task to include shadowing, which was included in order to increase demands upon the anterior attentional network, there was no longer evidence of RVF/LH inhibition. It was concluded that the LH inhibitory effects were mediated by the anterior attention system as the shadowing manipulation nullified these effects.

Regarding the CSM, a candidate LH area that might generate both inhibitory and facilatory control over activated semantic stores is the left inferior frontal sulcus (LIFS). The LIFS is of interest in this context due to recent fMRI findings, implicating this area in concept-based selection (see Kan and Thompson-Schill, 2004 for a review)^2^ and/or effortful processing (Makuuchi et al., 2009). Tensor diffusion imaging indicated that communication between the LIFS and left posterior cortical regions increased as a function of the difficulty of sentence processing (Makuuchi et al., 2009). Collectively, these studies led us to expect that attentionally induced facilitation and inhibition, such as that produced by the CSM, would be observed only in the LH.

The current study sought to examine whether the CSM might operate exclusively in the LH, and to test the CSM theory against competing, non-inhibitory theories by employing ERPs. The methods employed were a modified version of Barnhardt et al.'s (1996) procedure for producing negative priming (i.e., inhibition) on target words using weakly represented, newly acquired vocabulary items. The stimuli in the test phase were lateralized, thus permitting the exploration of possible differences between the hemispheres in the operation of inhibitory mechanisms. Two predictions were made for the RVF(LH) stimulation condition. It was predicted that targets that were related to, but not synonymous with, the preceding rare prime word, would be inhibited by the weakly activated primes. Inhibition would be evidenced by an increased negativity in the N400 region. Conversely, we predicted that synonym targets, which should fall within the “center” of the activation produced by the prime, would be facilitated. In this condition, facilitation would be evidenced by a reduced negativity in the N400 region.

Under LVF (RH) stimulation, no inhibition was predicted for any type of target. This prediction was made, first, because in the literature there is virtually no evidence of associative links between words in the RH. Hence, it can be concluded that there is no spreading of activation between items in the RH. For this reason, no CSM would be required. Secondly, according to previous research, the RH appears to lack the attentional capacity of the LH, which would be necessary to support the CSM. It was expected that synonym targets presented to the LVF/RH would evidence facilitation, in spite of the weak preactivation provided by the prime, due to the large number of overlapping features. Little or no facilitation was predicted for the non-synonymously related targets presented to the LVF/RH, the reason being that the number of features preactivated by the prime would probably not be sufficient, considering the weak activation level of the prime. The latter prediction partially emanates from our finding that overlap of a greater number of semantic features will create significantly more LVF/RH priming (Grose-Fifer and Deacon, 2004).

## Methods

### Participants

The ERP data reported herein were collected from 10 participants (four males and six females)^3^ between the ages of 18 and 32 (mean age = 21.9 years), who were pre-selected on the basis of a criterion, such that they had acquired the meanings of approximately 50% of the novel words presented during a learning phase (see below). Participants were recruited from posted advertisements and university classrooms and reimbursed at the rate of $10 per h. They were monolingual, native speakers of American English. English was the only language spoken in their homes. Participants were deemed to be right handed, with a mean laterality quotient of 89.92, according to the Edinburgh Handedness Inventory (Oldfield, 1971). None reported any history of medical/neurological disorders, or learning disabilities. Far point visual acuity, assessed at 10 feet with a Graham Field eye chart, was determined to be 20/20 in all but one subject, who evidenced 20/25 visual acuity. The latter individual was able to correctly read 10/10 practice stimuli presented on the stimulus display screen under the experimental conditions (see below). Written informed consent was obtained from all participants prior to their participation.

### Stimuli

The stimuli were English words, presented in black, on a white background. Words were 1 cm high, an average of 6.29 letters in length, and were centered 4 degrees to the left or right of a fixation mark (+). The primes were 104 rare English words. The majority of these (100 items) were selected from a larger corpus of rare words used by Barnhardt et al. Four additional rare words were chosen by the experimenters in order to permit words to be balanced for frequency and length across conditions. The targets were 600 common words, which were either synonyms of, related to but non-synonymous with, or unrelated to the prime. Additional targets were also added to the Barnhardt et al. stimuli to pair with the four additional rare words. Both the rare words and targets were found using Microsoft Bookshelf (1998 version), and Roget's Thesaurus (Roget and Browning, 1986). The relatedness of these additional stimuli was determined by the authors using the latter sources, and verified by other members of the research team. Because of the inherent rarity of the added prime words, they were not available in published norms of frequency or association. For all words, the mean number of letters and frequencies were fully balanced between conditions of Priming, Target type (synonyms and nonsynonymous related words) and VF for each class of common words. Word frequency was determined using the standard frequency index of Carroll et al. (1971). Examples of rare words, synonyms, and non-synonymous related words are presented in Table 1. On 80% of trials, a rare word prime appeared, followed by one of the three classes of targets (i.e., synonyms, related nonsynonymous or unrelated). One-half of the corpus of target words constituted the primed trials, composed of synonyms and non-synonymous related words (50% each) and the other half of unprimed trials. The trials were presented randomly and equiprobably to either the LVF or RVF. On the remaining 20% of the trials, which were not included in the analysis of ERP trials, either the prime or the target consisted of common words that could be classified as weapons (e.g., “SPEAR”). The other stimulus on these infrequent trials was either a rare word or a legal pseudoword, (e.g., “MIZZY”). These trials were included for use in a behavioral task, in order to engage sufficient semantic processing of the words, but were of no experimental interest. Four runs were presented, of 125 trials each. There were 50 trials per condition.

**Table 1:** **Examples of rare words, synonyms, and related words used as stimuli**.

**Rare words**	**Definitions**	**Synonyms**	**Related words**
Cark	To be anxious	Fret	–
		Worry	–
Blet	Overripe	Rotten	–
		Spoiled	–
Fadge	To do well at something	Achieve	–
		Excel	–
Krang	Whale meat	–	Blubber
		–	Gristle
Nubbin	A small ear of corn	–	Peas
		–	Beans
Shawm	Old-fashioned oboe	–	Bassoon
		–	Clarinet

### Procedure

#### Learning task

Rare English words were paired with a brief (1–7 word) definition (see Table 1). Each of the 104 rare words and their definitions were displayed in black on a white background for 7 s. The words appeared in 54 point New Times Roman font 3.75 cm from the top of the screen, horizontally centered, and the definitions appeared 3.75 cm from the bottom of the screen, also horizontally centered. The subjects were instructed to learn the definition of each word as precisely as possible, in order to be able to recall the definitions after a short delay.

Immediately following the learning session, counting backwards by threes was engaged in, from a pseudo-randomly chosen four-digit number, for three minutes, in order to prevent rehearsal of any of the definitions in short term memory. During the subsequent recall test, each rare word was presented alone for 5 s, in a new random order, and the subject was instructed to recall the definition for that word as precisely as possible, and if they were unsure, to guess the definition. Subjects were required to provide all of the information provided in the definition. Definitions were scored as correct or incorrect immediately after each response. No feedback was given, unless an incorrect definition was produced, in which case the experimenter said “No.” Only first responses were scored, in order to avoid any ambiguities.

Those individuals who recalled less than 40 definitions were trained an additional time, with a new random ordering of words, and then re-tested following another three-minute delay/interference period. Some required an additional training session in order to meet the recall criterion. Only those who scored within approximately 12 definitions of the 50% recall criterion (range ~40–62 definitions) were included in the ERP experiment reported here. This criterion insured weak activation of the prime.

The ERP recording session was immediately followed by a multiple choice task in which the rare word prime appeared, on the computer monitor, followed by four possible definitions. The choices consisted of: (1) the correct definition, (2) the definition of another rare word that had been previously studied, or (3) two definitions of words that had not been studied, but were similar in length and complexity to the rare word definitions. Participants responded by marking the appropriate choice on a response sheet at their own pace.

#### Eye movement calibration, and visual fixation training

Prior to presentation of the experimental trials a record was produced of the amplitude and morphology of each subject's visual saccades, which was later used as a basis for identifying and excluding trials containing these artifacts. A fixation cross appeared for 250 ms, followed by a pause of 250 ms. Words were then presented for 185 ms, four degrees to the left or right of fixation. Participants were instructed to move their eyes, and track the occurrence of each word, so that they could read it normally, while remaining still.

Following EOG calibration, participants were again shown lateralized words, but this time were instructed to read the words while maintaining fixation on the central mark. The horizontal EOG was monitored, and if any deviation from the fixation mark occurred, participants were told, “Keep your eyes on the mark.” The training phase was repeated until the participants could comfortably maintain a central fixation while reading the words.

#### Task performed during ERP recording

Trials began with the appearance of a fixation mark in black, against a white background, which remained on for the entire trial. Each trial began with a rare word prime stimulus, presented for 250 ms, followed 2050 ms later by a common word target, for 250 ms. The prime and target were always presented to the same VF. After a delay of 750 ms, a question mark appeared that lasted for 1000 ms. Trials commenced 1.5 s after the disappearance of the question mark. Participants were told that words would be presented on the computer screen, and that some of the words would be the new words that they just learned, while others would be common words that they already knew. They were instructed to read the two words, and make a decision as to whether either of them would normally be considered to be a weapon or an instrument of war. Following the question mark prompt, they were to use their right (dominant) hand to press the left mouse button if neither word was a weapon, and the right mouse button if either of the words was a weapon.

#### ERP recording procedures

The electroencephalogram (EEG) was recorded from 18 electrodes (FP1, FP2, Fz, F3, F4, C3, Cz, C4, P3, Pz, P4, T3, T4, T5, T6, O1, Oz, and O2) referenced to the tip of the nose. Horizontal electro-oculogram (EOG) was recorded from two electrodes, placed laterally to the outer canthi of the right and left eyes. Vertical EOG was recorded by electrodes on the supra and infra-orbital ridges of the left eye. Impedances for all electrodes were kept below 5 kOhms. The EEG and EOG were filtered on-line with a 60 Hz hardware notch filter, and off-line with a bandpass filter of 0.1–20 Hz. Any trial that contained horizontal eye movements was removed by hand from the continuous data file to ensure that only the data from properly lateralized stimuli were included. The horizontal eye movements were identified with the aid of the data recorded during the eye movement training and calibration phase described above. The continuously recorded data file was epoched from −200 ms before, to 3000 ms after the onset of the prime, at a rate of 456 Hz, resulting in 1459 data points per epoch. Files were baseline corrected on the basis of the 200 ms prior to the onset of the first stimulus (prime), and corrected again from 200 ms before the second stimulus (target) for the purpose of measurement and display. Trials with other electrical artifacts were removed first by hand, after which each file was checked automatically by an artifact rejection algorithm set to +/− 75 μ V, primarily to eliminate blink related vertical EOG activity.

#### Data analysis

Responses to the “weapon/non-weapon” judgment task were sorted as being correct or incorrect and mean percentage correct scores were tabulated.

As there were no large differences in the latency of N400 across conditions, a single measurement window was employed. The N400 was measured as the mean activity within a window extending from 380 to 515 ms after the target (S2). This was the narrowest common window where the N400 appeared in the grand means across electrodes and conditions. A four-way repeated measures ANOVA was performed with factors of Visual Field (left, right), Priming (unprimed, primed), Target Type (related non-synonymous, synonym), and Electrode (F3, Fz, F4, C3, Cz, C4, P3, Pz, P4, T3, T4, T5, T6, O1, Oz, O2). Note that Fp1 and Fp2 recording sites were dropped prior to analysis. Three-way ANOVAs were carried out for each VF separately, in order to investigate whether the nature of the interaction was as predicted, three-way ANOVAs were conducted using factors of Priming (unprimed, primed), Target Type (related non-synonymous, synonym), and Electrode (16). These analyses were followed up by two-way comparisons using the factors of Priming (2) and Electrode (16). The Greenhouse-Geisser correction was applied where appropriate. Partial eta squared (h^2^_*p*_) is provided as a measure of effect size for the comparisons of interest. It would have been of interest to sort the physiological data according to whether the definition of the novel prime was recalled or not. However, this was not feasible due to the number of trials that would have been required to construct an ERP of each type of trial.

## Results

### Behavioral data

Overall, the correct “weapon/non-weapon” judgment was made on 77% (*SD* = 5.8) of the trials. The participants provided an average of 50.7 (*SD* = 12.3), or 48.8% of the correct definitions on the recall test following their final training session (after the distraction task), thus achieving the goal of learning the rare word primes to the point where they would be weakly activated. In the multiple choice definition recognition task, which followed ERP recording, the average recognition score was 93.8 items (*SD* = 15.5) or 90.2%. Therefore, although the primes were weakly activated during the experimental task, the majority of the words achieved a level of representation sufficient to allow recognition of their definitions. It should be noted that performance on recognition tasks is most often better than performance on a recall task involving the same materials. As the definitions of the rare words were not shown between the learning phase and the post-recording recognition test phase, continued learning of the definitions seems unlikely.

### Physiological data

**Figure 3:**
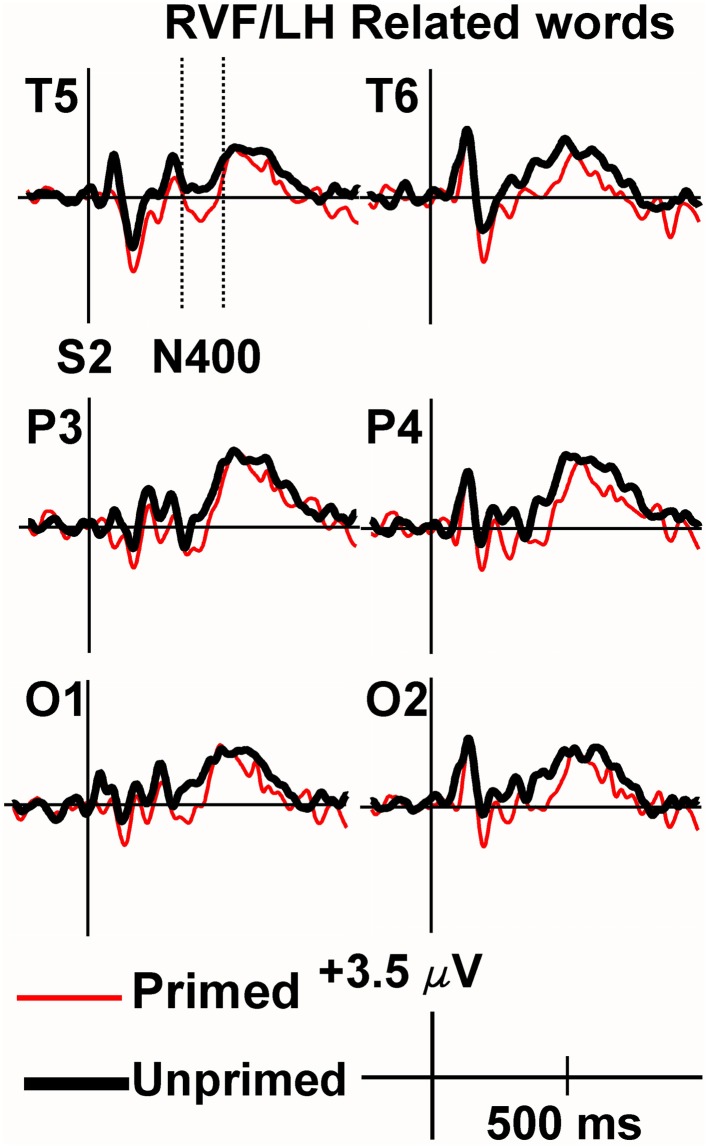
**Grand average ERPs recorded to non-synonymously related targets presented to the RVF/LH**. Non-synonymously related words produced more negative N400s than unrelated words (i.e., demonstrating inhibition).

**Figure 4:**
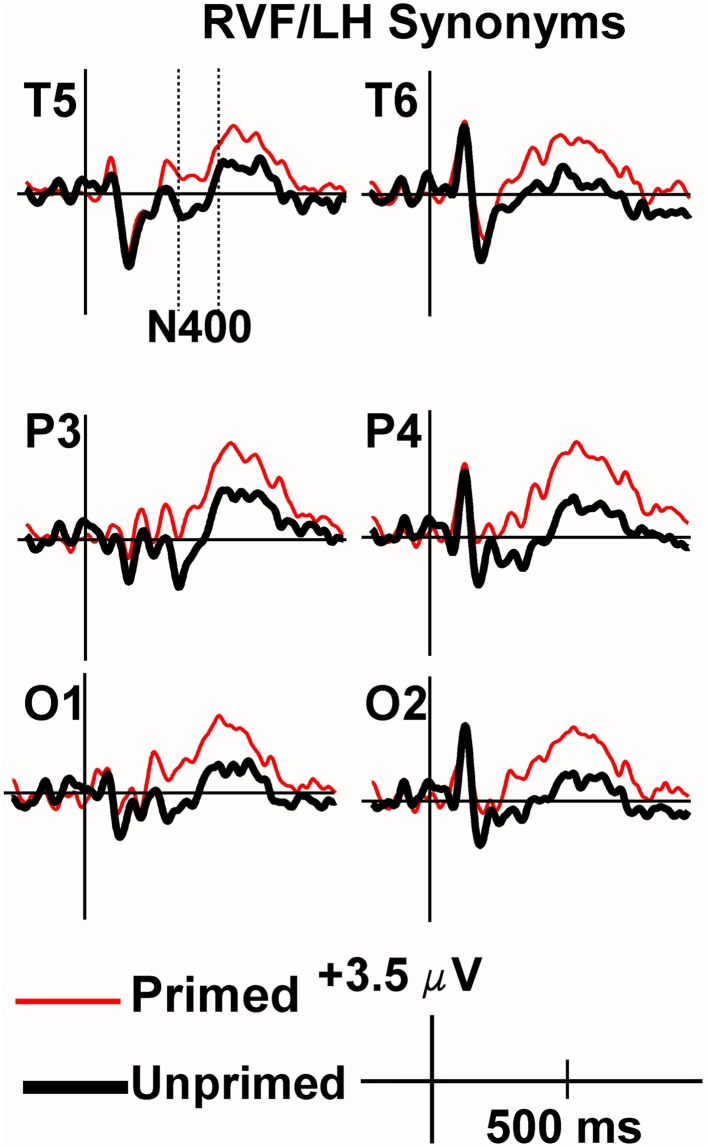
**Grand average ERPs recorded to synonym targets presented to the RVF/LH**. The Primed condition is printed in red, and the unprimed in black. Baseline correction has been performed from the target (the second stimulus in each trial). The N400 was significantly more positive for synonyms of rare words than to unrelated words (i.e., demonstrating facilitation).

**Figure 5:**
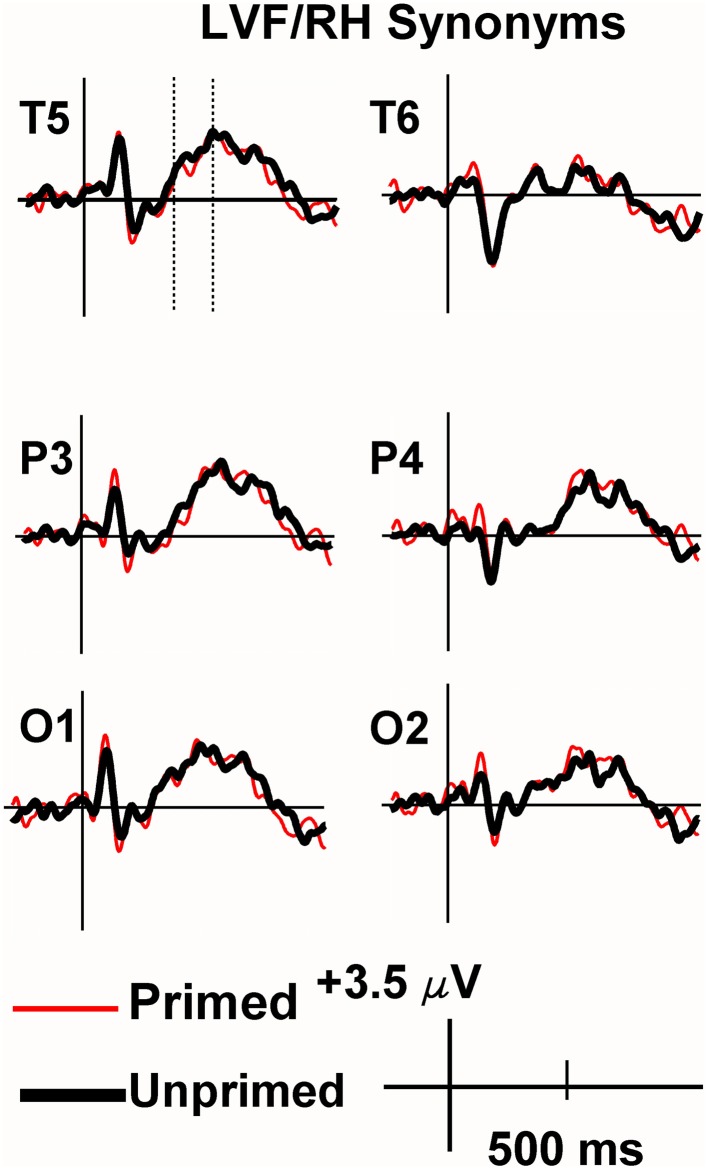
**Grand average ERPs recorded to synonym targets presented to the LVF/RH**. Synonym targets produced neither significant priming nor inhibition effects in the LVF/RH.

**Figure 6:**
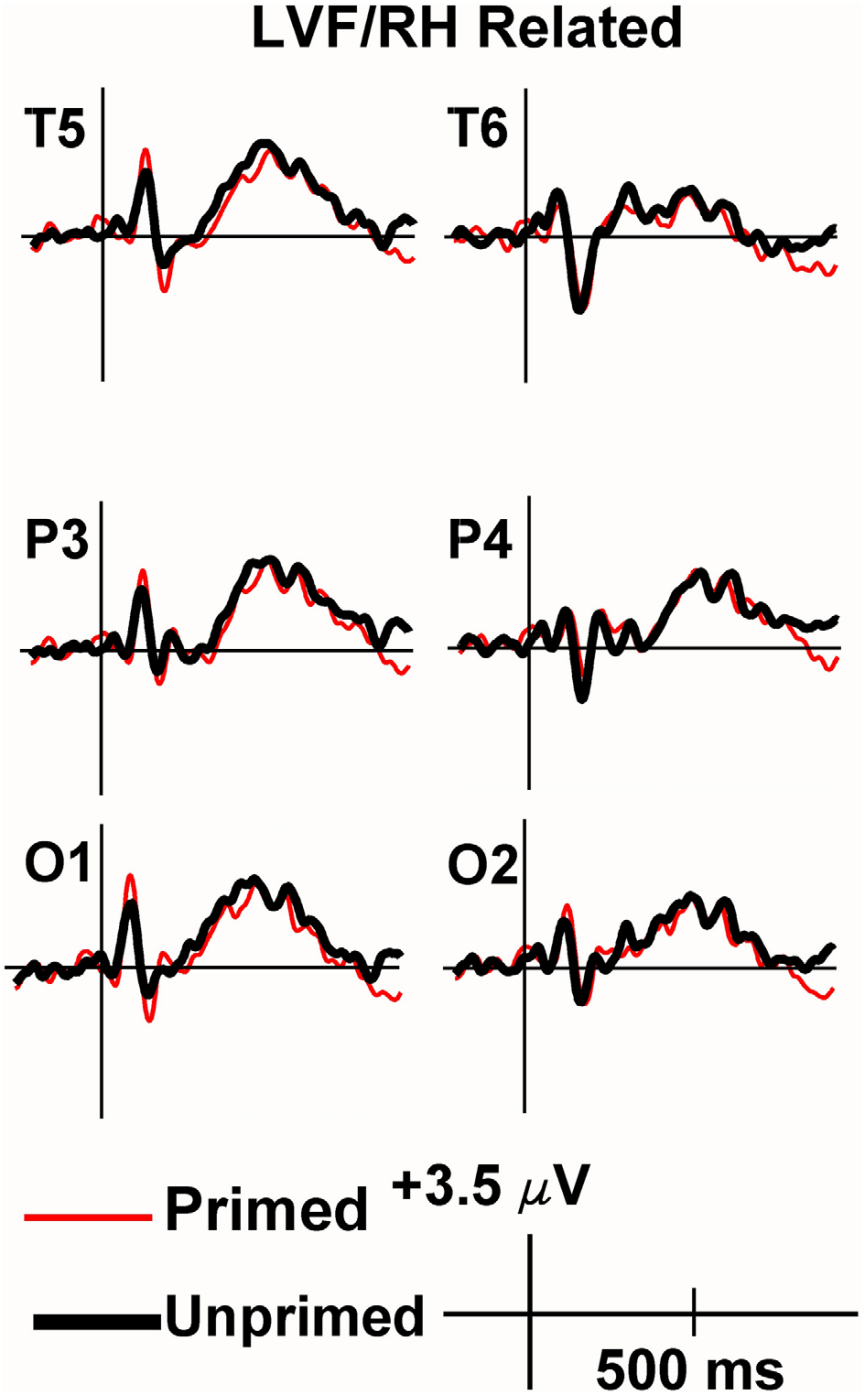
**Grand average ERPs recorded to non-synonymously related targets presented to the LVF/RH**. There were no significant inhibition or facilitation effects.

Viewing the grand average waveforms, the ERPs elicited by non-synonymously related items contained a larger N400 than those elicited by unprimed targets in the RVF/LH condition (Figure 3). Conversely the N400 elicited by synonyms in the RVF/LH condition was smaller (less negative) than that elicited by unprimed targets (Figure 4). In the LVF/RH condition neither synonym nor non-synonymous targets produced observable differences on the N400 (Figures 5, 6).

In the overall, four-way ANOVA, this produced a significant VF by Target Type (synonym vs. non-synonymous) by Priming interaction, *F*_(1, 9)_ = 5.75, *p* = 0.04, (*h*^2^_*p*_) = 0.39. Also obtained were Target Type by Priming *F*_(1, 9)_ = 9.19, *p* = 0.01, (*h*^2^_*p*_) = 0.51 and VF by Electrode, *F*_(15, 135)_ = 7.29, *p* = 0.004, (*h*^2^_*p*_) = 0.45 interactions.

In order to investigate the VF by Target Type by Priming interaction, three-way ANOVAs were carried out for each visual field separately. There was a Target Type by Priming interaction *F*_(1, 9)_ = 19.86, *p* = 0.002, (*h*^2^_*p*_) = 0.69 for the (RVF-LH), and a main effect of Electrode [*F*_(15, 135)_ = 6.983, *p* = 0.003]. No other interactions were significant for the RVF/LH. The same Target Type by Priming interaction was not observed in the RVF/LH. In fact, it was very far from significance in the LVF/RH [*F*_(1, 9)_ = 0.001, *p* = 0.99]. Other than a main effect of Electrode [*F*_(15, 135)_ = 10.64, *p* = 0.000], no main effects or interactions were significant for the LVF/RH condition. The main effect of priming did not even remotely approach significance at [*F*_(1, 9)_ = 0.05, *p* = 0.829]. Therefore, no further comparisons were performed on the LVF/RH data.

For the RVF-LH, two-way ANOVAs to compare each Target Type with the unprimed condition revealed that priming was significantly facilatory for synonyms *F*_(1, 9)_ = 10.95, *p* = 0.009, (*h*^2^_*p*_) = 0.55. The N400s to primed targets were less negative (*M* = 0.99 μV, *SEM* = 0.77) than those elicited in the unprimed condition (*M* = −1.16 μV, *SEM* = 0.91). The same Two-Way ANOVA performed upon the non-synonymously related words, however, demonstrated significant inhibition, *F*_(1, 9)_ = 6.39, *p* = 0.032, (*h*^2^_*p*_) = 0.42, in that the N400s were actually more negative in the primed condition (*M* = −0.79 μV, *SEM* = 0.61) than in the unprimed condition (*M* = 0.43 μV, *SEM* = 0.70).

Effect sizes were measured by *h*^2^_*p*_,which was interpreted according to the tables of Cohen (1992) in which 0.0099 is deemed to be a small effect, 0.0588 a medium effect and 0.1379 a large effect. Thus, the obtained effect sizes of 0.39 for the predicted overall three-way interaction, 0.55 for the LH facilitation effect, and 0.42 for the LH inhibition effect were all ample effect sizes indeed.

## Discussion

The main interests of the study concerned the possible operation of a CSM, its locus of operation in the LH, and its inhibitory nature. There were four main predictions. These were that we would obtain (1) significant facilitation from synonyms in the RVF/LH, (2) significant inhibition from non-synonymous related words in the RVF/LH, (3) no significant inhibition from non-synonymously related words in the LVF RH, and (4) significant facilitation from synonyms in the LVF/RH.

The first three of the predictions were borne out. A significant VF by Target Type by Priming interaction was obtained on the amplitude of the N400. Subsequent analyses indicated that under RVF/LH stimulation, newly learned rare word primes produced facilitory N400 effects for synonyms, and inhibitory N400 effects for non-synonymously related targets. The statistical measure of effect size (*h*^2^_*p*_) indicated a very large effect for each of the latter comparisons (see results section for interpretation). Conversely, LVF/RH stimulation elicited neither significant facilitation nor inhibition from synonymously or non-synonymously related targets. Thus, as expected, the RVF/LH ERP data provided physiological evidence of a CSM that was convergent with the behavioral data reported by other investigators (Dagenbach et al., 1989; Carr and Dagenbach, 1990; Barnhardt et al., 1996; Wentura and Frings, 2005; Frings et al., 2008).

The presence of significant inhibition in the LH supports our view that the LH semantic system operates on the basis of a spreading activation system, comprised of bound representations of items (Figure 2) wherein activation would spread via associative links. In a spreading activation system unbridled facilitation between associated items would reduce the accessibility of weakly learned items. It was predicted that the weakly activated rare word primes would inhibit processing of non-synonymously related targets, and facilitate the analysis of synonyms. In the LH, the CSM does, indeed, appear to have functioned as would have been expected, inhibiting more distantly related targets, and facilitating the synonym targets.

We predicted no inhibition under LVF/RH stimulation, following from our previous data and other reviewed literature suggesting differential processing of associations and features in the two hemispheres. Again, since there is no associative priming in the isolated RH this suggests that the RH semantic system is subserved by a strictly feature-based distributed system. As there is no item-to-item spreading of activation, there would be no requirement for the CSM to constrain such activation. In line with the predictions, there was no inhibition from the rare word primes in the LVF/RH data. The absence of RH inhibition was, thus, consistent with this laboratory's contention that the RH represents words on the basis of distributed features.

The data are also consistent with the model of Burgess and Simpson (1988), to the extent that the latter authors maintained that inhibition is generated exclusively within the LH, whereas the RH does not share this capacity. However, these authors would not have predicted inhibition from words related to weakly learned primes but facilitation from their synonyms. The coarse coding model (Beeman, 1998) would not have predicted inhibition, but rather passive withdrawal of resources, in which case reversed priming would not have been obtained. In Chiarello's earlier work she was of the opinion that inhibition could be produced only in the LH. This position appears to have been reversed in a later study where foveally presented distractors produced effects on RH targets (Chiarello et al., 1995). A reviewer of this paper brought our attention to the fact that a simulation based upon mediated priming data^4^ (Brunel and Lavigne, 2009) predicted inhibition effects that were in the same direction as in the present study. We feel compelled to note, however, that the amount of inhibition predicted was extremely small in all conditions (from their figures; approximately 5–7 ms at an 800 ms SOA, 3–4 ms at a 400 ms SOA, and 1–2 ms at a 200 ms SOA). At the 400 ms SOA their model predicted that the inhibition effects would be larger in the LH, but the difference was also exceedingly small (about 2 ms).

From a neuroanatomical perspective, the restriction of a highly significant inhibition effect to the LH in the present study is consistent with fMRI studies reviewed by Kan and Thompson-Schill (2004). The latter fMRI studies implicated the left inferior frontal gyrus in conceptual selection in single word paradigms. Conceptual here refers to meaningful representations of words or objects.

Under LVF/RH stimulation, there was no significant facilitation from the partially learned primes to their synonyms, or to related non-synonymous items. We had expected that the synonym targets, in particular, might have evidenced some degree of facilitation due to semantic feature overlap, even though the primes were weakly activated. One explanation for why the weak primes did not create priming on the targets is that the long SOA employed might have allowed differential decay in the two VFs. Our previous experiments demonstrated feature based priming under LVF/RH stimulation, using shorter SOAs (250 ms) than in this study (2300 ms). The long SOA was adopted in order to allow inhibition to accrue (Neely, 1977), and also allowed us to essentially replicate Barnhardt et al. (1996) in a manner that was amenable to ERP recording using the split VF technique. While this laboratory has found evidence of both controlled and automatic priming outlasting 2 s under central presentation (Deacon et al., 1999), the decay rates of activation in the two hemispheres have not been studied under conditions where both prime and target are lateralized. It is, therefore, possible that the activation of semantic codes decays more rapidly in the RH.

Alternatively, it is possible that a critical amount of encoding might not have taken place in the RH to produce priming on the targets. While our pretest measure of verbal recall indicated partial knowledge of the word meanings, the test stimuli were presented foveally in this phase of the experiment. The learning and post-test phases were conducted using central presentation due to our uncertainty as to how many trials, and hence, how long a testing session would be required for each subject to learn the meanings of the words. Subsequently, however, there were no LV/RH effects of priming when the ERP stimuli were lateralized, in spite of significant LH facilitation and inhibition.

As priming was produced under RVF/LH stimulation within the same runs, the task requirements appear to have been sufficient to engage the participants to process the meanings of the primes and targets in both hemispheres. Moreover, our previous studies have found statistically significant priming under LVF/RH stimulation using very similar stimuli and viewing conditions. Given that the perceptual requirements of the present experiment were equivalent to those in our earlier studies, where priming occurred in both VFs/hemispheres, it would appear that the novel words were not sufficiently encoded in the RH to create priming.

Regarding other models, both the coarse coding hypothesis and Chiarello's theoretical framework propose that the RH is able to maintain more weakly activated codes than the LH. In this regard, the point of view of Yochim et al. (2005) is also akin to the coarse coding model. Yochim et al. speculated that reciprocal activation and inhibition across hemispheres could be used to fine tune semantic fields. The model of Brunel and Lavigne is likewise similar to the coarse coding hypothesis and Chiarello's framework as it also suggests that the RH can maintain more activated material simultaneously. Chiarello's framework, the views of Yochim et al. and the model of Brunel and Lavigne would have predicted greater facilitory priming in the RH than in the LH, and are thus, not totally consonant with the current data.

The present physiological data, consisting of an inhibitory effect observed directly upon the N400, argue against alternative, non-inhibitory explanations of the CSM. Kahan (2000) suggested that when a prime is weakly activated, target processing is not inhibited, but rather, that the response to the target is delayed because the analysis of the weakly activated prime is not yet completed. Kahan arrived at these conclusions using masked stimuli. Specifically, Kahan argued that when a masked word is presented that is difficult to identify, the target is used to aid in the identification of the prime under conditions in which the subject expects a predictable relationship between the prime and target. These conclusions were primarily based upon an experiment that incorporated more repetition than semantic priming trials within the same run (see Experiment 2). Semantic primes produced facilitation of RT, whereas repetition of the prime produced inhibition. These results would not have been predicted by the CSM theory, in that repeated words should always fall within the center of the mechanism, and thus could not be inhibited, whereas distantly related items should have fallen in the surround and been inhibited. The pattern of results was essentially the opposite to that reported in several studies that have investigated repetition and semantic priming with the goal of examining the CSM (Carr and Dagenbach, 1990; Wentura and Frings, 2005; Frings et al., 2008). These particular studies provide a basis for comparison, in that the primes were masked, repetition and semantic priming were manipulated within the same runs, and a behavioral response to the prime was employed in order to sort the RTs to the target.

A main distinguishing aspect of Kahan's experimental design was that the proportion of repeated stimuli was much higher than in other studies. The higher proportion of repeated trials was included in order to encourage the strategy of post-lexical matching of the prime and the target. One caveat, which he acknowledges, is that manipulating strategic effects on RT is inconsistent with the masked stimuli being dissociated from consciousness. This, in turn suggests that the masked stimuli used by Kahan were more perceptible, and therefore not as weakly activated as in other studies. There was no threshold-setting task in Kahan's second experiment. A difference in the accessibility of the primes might, therefore, have allowed post-lexical processes to operate that were not present in other behavioral masking studies.

The data reported here are not interpretable as resulting from post-lexical matching, as the N400 was used as an index of both facilitation and inhibition. Since the case has been made that the N400 is not affected by post-lexical matching processes (see Introduction), the data provided relatively strong evidence of the inhibitory nature of the CSM in the LH.

Several previous behavioral studies concerning the CSM based their conclusions on the analysis of error trials, whereas we have found a measurable ERP CSM effect by considering all trials, irrespective of whether the definitions of the novel words were recalled during the learning phase. The inclusion of all trial types, coupled with evidence of learning in the RVF/LH, where the CSM was active, lends further ecological validity to the CSM. The mechanism would appear to assist in the acquisition of language, and the retrieval of word meanings. While the present study employed weakly activated, newly acquired words, somewhat similar N400 data have been obtained using well-known words. The N400 was more negative (i.e., inhibited) for well-known words with larger orthographic neighborhoods (Laszlo and Federmeier, 2011), than those with smaller neighborhoods, suggesting that the CSM might function under ordinary circumstances as well, in response to increased spreading activation and a resultant decrease in the signal to noise ratio.

The methods used in other studies of word learning have varied widely and are all considerably different from those used in the present experiment. No studies that we are aware of lateralized the stimuli, nor were design elements incorporated to examine inhibition from the newly acquired words. In spite of methodological differences, several other studies found evidence consistent with the presence of inhibitory processing of newly acquired words, when the novel words were not learned in the context of sentences. Perfetti et al. (2005) trained subjects over a 45-min study session using very similar methods to the present study. As testing for a CSM was not the purpose of the Perfetti et al. study, there was no performance criterion set for inclusion. As would be expected, the N400 to trained novel words was significantly more negative than to familiar words. Consistent with our findings, however, the N400 elicited by trained words was also more negative than that elicited by untrained words. Without first training subjects, ERPs were recorded to novel words by Frishkoff et al. (2009) during a lexical decision task. Two late negativities were elicited by the novel words, an N2c and an N350. The N2c was significantly more negative when elicited by novel than familiar words. These data were also consistent with those reported here to the extent that the modulator of the N2c was localized to the LH. Other data that might be interpreted as evidence of inhibition have been acquired from 14 month-old infants (Friedrich and Friederici, 2008). Consistently pairing an auditorily presented non-word with an image of a non-object produced a larger amplitude N400 for congruous than incongruous pairings. It is difficult to determine, however, whether the usual N400 congruity effect was reversed in infants due to the memory of the newly acquired words being weakly activated, or other factors related to the immaturity of the developing nervous system.

By contrast, learning new words within the context of sentences appears to produce facilitory changes upon the N400, without obvious evidence of inhibition. When second language word learning was assessed by recording ERPs, at three intervals, over a 14-h period of classroom instruction, the usual N400 priming effect was found for trained, but not naive subjects (McLaughlin et al., 2004). In the trained group of subjects, a difference was also obtained between second language words and pseudo words. A number of other studies have also reported rapid learning in experimental paradigms where novel pseudo words were embedded in context (Mestres-Missé et al., 2007, 2010; Borovsky et al., 2010, 2012; Batterink and Neville, 2011). Borovsky et al. found significant effects attributed to learning only when the sentences were highly constraining.

Surveying the findings of the contextual learning studies the commonality that emerges is that these studies have all reported changes in the N400 that are consistent with facilitation. There do not appear to be data interpretable as inhibition. The findings reported in the studies in which no context was provided were very different. There is some tentative evidence of inhibition in these studies. The difference between the two groups of studies could have been that the constraint provided by the sentence structure, when provided, may have allowed facilitation to occur, rather than inhibition. In the CSM framework, inhibition is necessary in order to dampen competing lexical items, when newly learned words are retrieved. Essentially, the CSM would not have been necessary to invoke if the meaning was already tightly constrained by the sentence context in which the word is learned in, and the sentence was retrieved with the word.

In summary, the data provided physiological evidence for the existence of a CSM. As per the conclusions of Dagenbach and colleagues, the mechanism indeed appears to involve inhibition. The study, further, implicated the LH as the locus of the inhibitory effect, consistent with the existence of a spreading activation system of semantic representations in LH. The CSM may offer an explanation for the puzzle of how the brain can simultaneously acquire new vocabulary rapidly, and maintain existing semantic representations with a minimum of interference. The data derived using the present methodology, however, suggest that the isolated adult RH is, perhaps, less adept at acquiring semantic information pertaining to new vocabulary, in the visual modality, over very short training periods.

### Conflict of interest statement

The authors declare that the research was conducted in the absence of any commercial or financial relationships that could be construed as a potential conflict of interest.

## Footnotes

^1^Threshold-setting tasks are used in these experiments in order to bias the subjects processing strategy. The threshold-setting task induces subjects to process the words in either a shallow or deep manner. The assumption is that the same depth of processing used in the threshold-setting task will carry over into the next phase of the experiment. Thresholds for responding to weakly activated stimuli are biased by the preceding threshold-setting task.

^2^Concept refers here to the long-term representations of anything meaningful, including words or the objects that they represent.

^3^Although sufficient ERP trials were collected from two additional participants to allow analysis, their data are not reported on here as they performed below chance on the semantic judgment task.

^4^We were asked to comment upon Brunel & Lavigne's simulation but are not sure how appropriate the comparison is. Their simulation was based upon data from a mediated priming study. This type of study does not use words that are newly learned or weakly activated in any other way. Although the prime is more distantly related to the target in mediated priming, than in direct priming, all the words involved are firmly instantiated in long-term memory (e.g., LION-STRIPES with tiger being the mediating concept).
