# Supplementary material for: Electrophysiological evidence for the action of a center-surround mechanism on semantic processing in the left hemisphere
Source: Front Psychol. 2013 Dec 30;4:936. doi: 10.3389/fpsyg.2013.00936 (PMC3874853; doi:10.3389/fpsyg.2013.00936)
Supplement: Supplementary file 1 [file DataSheet1.DOC]

| **List 1** | | **List 2** | |
| --- | --- | --- | --- |
| **RAREWORD** | **TARGET** | **RAREWORD** | **TARGET** |
| BATRACHIAN | AQUATIC | KEET | HEN |
| GRIGNET | GROUSE | KECK | ACCELERATE |
| FIGULATE | RECUR | ANCHORET | ENDOCRINE |
| FOULCHER | KNAPSACK | THEW | BOW |
| SHOGGLE | TEARGAS | CORUSCATE | HELIUM |
| SHOGGLE | MISSILE | BRISANCE | PASTEL |
| ROKER | FRUIT | KECK | PUKE |
| FEEK | ROAM | COZEN | OBLIGE |
| SCRUMP | POISON | ANCHORET | CHANCELLOR |
| ORISON | BLESSING | COARCT | RESTRAIN |
| REPKIE | MERCURY | LONK | GOAT |
| ROKER | YARDSTICK | SPEAR | MIZZY |
| UPEYGAN | TRICERATOPS | MOPUS | PENNY |
| CLAW | SHOGGLE | A-BOMB | RABIATOR |
| HISPID | CHESS | BEVER | WRESTLER |
| BATRACHIAN | AMPHIBIAN | HAPTIC | MARTINI |
| BETON | ROLLERSKATES | SORNER | TORPEDO |
| LONK | SCARE | BEVER | REFRESHMENT |
| CURPLE | BOTTOM | THRAWN | PEROXIDE |
| CLAVIGER | AIR | RAZOR | RABIATOR |
| SCRUMP | HATCHET | FENERATE | CHOCOLATE |
| MINEFIELD | SCUDDICK | ANICULAR | FOOLHARDY |
| DRECCHE | HAG | COARCT | MOLECULE |
| DRECCHE | ANNOY | CORYZA | APRICOT |
| ELECTUARY | PILL | CHEWINK | ROBIN |
| DIGHT | ARRANGE | ELECTUARY | CAPSULE |
| NOOP | WORLD | CLAVIGER | MUSCLES |
| SCUDDICK | STICK | ACICULAR | POINTED |
| FEEK | BUTTE | THRAWN | ICEBERG |
| AGROTE | SWALLOW | CHEWINK | WORMS |
| SYRT | EYE | MOPUS | NICKEL |
| NUBBIN | RODENT | BIBBLE | PROPOSED |
| TRESAYLE | BANKS | ALFIN | ROOK |
| KECK | VOMIT | RABIATOR | SICKLE |
| STILETTO | XYSTUS | OLID | STENCH |
| IMBOSK | CLASSIFY | SORNER | ARTILLERY |
| TRUCKLE | SLINGSHOT | MOPUS | BITTERS |
| FOULCHER | DESTROYER | BRISANCE | BLAST |
| BOMB | TRUCKLE | BIFFIN | MANHATTAN |
| ENSKY | FABRIC | ROCK | THEW |
| IMBOSK | LIME | DRINTLING | MONITOR |
| ORISON | BOURBON | FENERATE | VINEGAR |
| ELRIG | GIRL | THRAWN | PRETZEL |
| FOULCHER | BACKPACK | COZEN | PERSUADE |
| VOTARY | ARROW | CORUSCATE | SHINE |
| NUBBIN | BEANS | COARCT | CONTROL |
| FEEK | CHAPERON | CHEWINK | OXYGEN |
| IMBOSK | UNIVERSE | OLID | BEDBUG |
| ORISON | HYMN | DRINTLING | CLUCK |
| HISPID | COURSE | POOKS | HAYSTACKS |
| DRECCHE | JUICE | GELOGENIC | FUNNY |
| EXPLOSIVE | VOTARY | CROSSBOW | SCRUMP |
| ELRIG | LAVA | CORUSCATE | SHIMMER |
| BETON | PAVEMENT | FENERATE | BORROW |
| BETON | CEMENT | GELOGENIC | COMICAL |
| VOTARY | SPEARGUN | MENSEFUL | PIPE |
| ENSKY | BLADDER | ACICULAR | NORFOLK |
| LADDLE | DUCHESS | BIBBLE | HELENA |
| BAR | SCUDDICK | BRISANCE | CONCUSSION |
| ENSKY | CANNONIZE | MORTAR | MIZZY |
| SYRT | SWAMP | CLAVIGER | CUSTODIAN |
| DIGHT | RENTAL | BIBBLE | DRUNK |
| AGROTE | SECTIONED | KEET | CHICKEN |
| DIGHT | ORGANIZE | ACICULAR | BANDAGE |
| NUBBIN | BASEMENT | CHEWINK | LARK |
| GRIGNET | PHEASANT | ACICULAR | SHARP |
| BATRACHIAN | POOL | BEVER | SNACK |
| AGROTE | OVEREAT | OLID | STINK |
| HALSEN | PROPHECIZE | SHAWM | CLARINET |
| TRESAYLE | NEPHEW | COARCT | URANIUM |
| SYRT | ORGANIC | ANICULAR | HOTEL |
| FIGULATE | PORCELAIN | COZEN | CONVINCE |
| UPEYGAN | BASE | NAIL | THINGUS |
| ORISON | EXHAUSTION | BIBBLE | ALCOHOLIC |
| BATRACHIAN | WARSAW | BRISANCE | EXTENSION |
| TRUCKLE | DAGGER | DRINTLING | NEBULA |
| HISPID | CANARY | MIZZY | PISTOL |
| ROKER | XENON | HAPTIC | RUB |
| SCUDDICK | RIFLE | KEET | FISCAL |
| UPEYGAN | JUSTICE | BLET | SPOILED |
| HISPID | BUMPY | BLET | HICKORY |
| HALSEN | PREDICT | COZEN | WIPER |
| REPKIE | STARFISH | ANCHORET | HERMIT |
| CURPLE | MITE | HOSE | THINGUS |
| FEEK | RAMBLE | CORUSCATE | CARDINAL |
| LADDLE | COUNTESS | RABIATOR | BLUDGEON |
| HALSEN | FLY | HAMMER | SORNER |
| CURPLE | DINOSAUR | ANICULAR | IDIOTIC |
| ELRIG | ESCALATOR | ALFIN | GOD |
| GRIGNET | JUGGLE | CORYZA | FLU |
| FIGULATE | GRATER | SHAWM | BASSOON |
| IMBOSK | CONCEAL | ALFIN | PAWN |
| ENSKY | IDOLIZE | POOKS | MISSIONS |
| UPEYGAN | HIPPO | THEW | PIN |
| REPKIE | JELLYFISH | GELOGENIC | OFFICER |
| CURPLE | ASS | KEET | QUARTZ |
| FOULCHER | MEDIUM | MOPUS | CONCORD |
| HARPOON | SHOGGLE | ANICULAR | LIGHTNING |
| THINGUS | CLEAVER | LONK | KETTLE |
| BETON | DROMEDARY | BAT | THEW |
| FIGULATE | POTTERY | CORYZA | COUGH |
| DIGHT | EGGPLANT | BIFFIN | CHERRY |
| LADDLE | LIVID | MIZZY | CROWBAR |
| NUBBIN | PEAS | THRAWN | GNARLED |
| MACHINEGUN | XYSTUS | BEVER | RAYON |
| LADDLE | YAP | BLET | GRIDDLE |
| KECK | NAVEL | GELOGENIC | CLIMBING |
| LONK | LAMB | HAPTIC | FINGERLIKE |
| TRESAYLE | AVALANCHE | ANCHORET | RECLUSE |
| SYRT | BOG | FENERATE | MORTGAGE |
| TRESAYLE | NIECE | ALFIN | BOUILLON |
| CLAVIGER | CLEANER | BIFFIN | PEAR |
| ELRIG | CHICK | BIFFIN | VENICE |
| REPKIE | ZOOLOGY | THINGUS | GRENADE |
| TANK | TRUCKLE | OLID | HOLLOWS |
| ELECTUARY | NOISE | POOKS | STRAW |
| AGROTE | GORGE | DRINTLING | GOBBLE |
| WHIP | VOTARY | ELECTUARY | DUCK |
| NOOP | KNEECAP | SHAWM | GUARD |
| NOOP | FREIGHT | BLET | ROTTEN |
| NOOP | FOREARM | HAPTIC | UMPIRE |
| GRIGNET | NOSE | CORYZA | WRUNG |
| ROKER | T-SQUARE | FIRE | SCRUMP |
| HALSEN | HORSES | SHAWM | IVAN |

| **List 3** | | **List 4** | |
| --- | --- | --- | --- |
| **RAREWORD** | **TARGET** | **RAREWORD** | **TARGET** |
| CRIBBLE | STRAIN | CARK | SAINT |
| NIKIN | GENIUS | DACOIT | THUG |
| CAXON | WIG | BULDERING | INVERT |
| SCION | VENTED | CHILLIAD | STONE |
| BANTLING | INFANT | DACOIT | SINKER |
| DAUT | EXPLODE | HUCKSUM | WAIST |
| CURTATE | PINEAPPLE | BURGOO | FAUN |
| APRICATE | TAN | COLUBRINE | TRAPDOOR |
| TROMBASH | HAIRY | SNUDGE | VEERS |
| PALETOT | HURRICANE | IRENIC | WART |
| AUBADE | ANALYZES | BRUMOUS | FOGGY |
| MARLISH | ARSENIC | FUSCOUS | NEGROID |
| REVOLVER | SORNER | ARENOSE | BEER |
| FISTS | SNASH | IRENIC | VIOLENT |
| SCION | PRINCESS | ISWONK | LABOR |
| NIKIN | CONFEDERACY | GUMPELFIK | SHOTGUN |
| NEEDLE | SNUDGE | ALEGER | POP |
| CRIBBLE | LICE | ISWONK | DOG |
| MARLISH | JAVELIN | CARFAX | NEXUS |
| NIKIN | DOLT | XYSTUS | TOMAHAWK |
| ILLTH | POVERTY | CARK | MINT |
| APRICATE | COUNTRY | DROMOS | TRAIL |
| ABROSIA | FAVORITE | ARENOSE | GRANULAR |
| ACCIPITER | MANTLE | SLOOM | AX |
| AUBADE | SONNET | EXCULPATE | ACQUIT |
| CRIBBLE | SIFT | WAPACUT | SHERIFF |
| PALETOT | SWEATER | EXCULPATE | VINDICATE |
| CRIBBLE | SWAN | TITBOW | ELEPHANT |
| PITCHFORK | SNASH | FUGACIOUS | FIBS |
| GLUNCH | SULK | SABER | GUMPELFIK |
| SNASH | CUDGEL | FUSCOUS | HACK |
| AURICOME | CANON | BRUMOUS | SCHOOL |
| WAPACUT | PARROT | GAS | SLOOM |
| NAPERY | COMMERCE | CHAIN | SINCIPUT |
| DORSOM | TEETH | IRENIC | TRANQUIL |
| AUBADE | BALLAD | BRUMOUS | BELLY |
| ABROSIA | FASTING | BLADE | SLOOM |
| GLUNCH | EXIT | BURGOO | HATE |
| SNUDGE | CANNON | CHILLIAD | BULLET |
| AURICOME | CLEANSE | GOETY | CLOCKS |
| SNASH | SCYTHE | EIDOLON | KATIE |
| IRON | MARLISH | ARENOSE | PAINTINGS |
| TROMBASH | DISCUS | CERULEAN | SECULAR |
| KEN | BASIL | ETTLE | PINNACLE |
| FADGE | EXCEL | SNUDGE | BEAMS |
| CURTATE | TUNNEL | FUSCOUS | NECTAR |
| BURGONET | RUG | SINCIPUT | THIRTY-EIGHT |
| COOM | CLOUD | ETTLE | ASPIRING |
| CURTATE | ABBREVIATED | MACE | SMATCHET |
| AUBADE | FONT | EIDOLON | SPIRIT |
| BIGGIN | DIESEL | FUGACIOUS | BRIEF |
| AURICOME | REDHEAD | BURGOO | CEREAL |
| BIGGIN | SCREEN | CERULEAN | AZURE |
| DAUT | NOTES | TITBOW | GLARE |
| ACCIPITER | EYELET | SINCIPUT | H-BOMB |
| CADGE | IMPLORE | ALEGER | BRAKE |
| CLAVER | DEAN | GUMPELFIK | KNUCKLES |
| CLAVER | CRYSTAL | TITBOW | GEOMETRY |
| APRICATE | FLEA | BAYONET | GUMPELFIK |
| GLUNCH | MIXER | COLUBRINE | DEVIOUS |
| FADGE | TOWER | CAXON | TUPE |
| SPIKE | MARLISH | CERULEAN | SKY |
| ABROSIA | BICEP | SNUDGE | GRINCH |
| BURGONET | HAT | CARFAX | PUREE |
| FADGE | PLUM | BURGOO | PORRIDGE |
| DAUT | CARESS | BULDERING | MUGGY |
| BIGGIN | ODDITY | BOOMERANG | JINK |
| PALETOT | SETUP | FUSCOUS | EBONY |
| COOM | DERVISH | COLUBRINE | SLY |
| ROCKET | SNUDGE | DROMOS | TRACK |
| CLAVER | CHAT | ISWONK | BRUSH |
| KEN | ENLIGHTEN | HENOTIC | RUDDER |
| TROMBASH | PEDAL | GOETY | WITCHCRAFT |
| KEN | NORMAN | BANTLING | TODDLER |
| DAUT | TOUCH | HUCKSUM | THIGH |
| BUNKUM | DOCTOR | ETTLE | MOTIVATED |
| FADGE | ACHIEVE | CAXON | DETAILS |
| COOM | WRY | DACOIT | RIBS |
| NAPERY | CURTAINS | CLUB | CHILLIAD |
| SMATCHET | CATAPULT | TITBOW | MIRAGE |
| SCION | PRINCE | SNUDGE | SCROOGE |
| WAPACUT | LITE | DORSOM | ORTHODOX |
| AURICOME | BRUNETTE | BULDERING | SWELTERING |
| ILLTH | RELISH | CARFAX | CROSSROADS |
| SAMBAR | ELK | EXCULPATE | SERVICE |
| FLAMETHROWER | MENSEFUL | CARK | FRET |
| BIGGIN | FILTER | KRANG | DALE |
| SNUDGE | SWORD | KRANG | GRISTLE |
| NAPERY | MOCCASINS | XYSTUS | KNIFE |
| KEN | TEACH | GOETY | VOODOO |
| DORSOM | UNSOLD | ETTLE | DARN |
| ACCIPITER | FALCON | KRANG | BLUBBER |
| MENSEFUL | BRICK | EIDOLON | FAWN |
| SCION | FREAK | HUCKSUM | NITROGEN |
| CADGE | FLOUR | DACOIT | CUTTHROAT |
| BURGONET | HEADGEAR | DORSOM | PALET |
| CURTATE | DIMINISHED | BRUMOUS | VAPOROUS |
| JINK | DYNAMITE | CARFAX | RAG |
| BUNKUM | FALSEHOOD | BULDERING | GENITAL |
| CADGE | ORLON | CARK | WORRY |
| APRICATE | BASK | ALEGER | HAPPY |
| CAXON | DONKEY | DROMOS | SANITY |
| TROMBASH | FRISBEE | GOETY | SAWHORSE |
| FANGS | JINK | CERULEAN | BUG |
| SAMBAR | TRANSPORT | MISSILE | CHILLIAD |
| SAMBAR | MOOSE | FUGACIOUS | BEET |
| GLUNCH | POUT | SLOOM | ROPE |
| CADGE | PLEAD | HENOTIC | FLUORINE |
| NAPERY | NAPKINS | ALEGER | JOYFUL |
| BANTLING | VAN | LANCE | SINCIPUT |
| COOM | NEURON | COLUBRINE | CATNIP |
| BURGONET | ELDER | ARENOSE | GRAINY |
| SMATCHET | BAZOOKA | KRANG | ZEPHYR |
| JINK | GUN | ISWONK | TOIL |
| DART | MENSEFUL | HENOTIC | MELODY |
| ILLTH | AFFLUENCE | LASER | SMATCHET |
| BUNKUM | DIRECTOR | HENOTIC | TUNE |
| NIKIN | LIZARD | HUCKSUM | SCALE |
| SAMBAR | BATHROOM | IRENIC | USHER |
| CLAVER | GOSSIP | FUGACIOUS | MOMENTARY |
| ILLTH | OLIVE | EIDOLON | GHOST |
| ABROSIA | STARVING | DROMOS | VOLT |
| PALETOT | JACKET | EXCULPATE | MISGUIDED |
